# Supplementary material for: Sensitivity to gait improvement after levodopa intake in Parkinson’s disease: A comparison study among synthetic kinematic indices
Source: PLoS One. 2022 May 12;17(5):e0268392. doi: 10.1371/journal.pone.0268392 (PMC9098031; doi:10.1371/journal.pone.0268392)
Supplement: Attachment — Submitted filename: Response to Reviewers.DOCX [file pone.0268392.s002.DOCX]

Dear Manabu Sakakibara,

please find enclosed a revision of our paper entitled “Sensitivity to gait improvement after levodopa intake in Parkinson’s disease: a comparison study among synthetic kinematic indices.” to be considered for publication in Plos One.

We thank the editor and the reviewers for their efforts in evaluating our manuscript. We did our best to follow the comments made by the reviewers, and hope that this revised submission will be adequate for publication.

In what follows, we firstly summarize the main changes, and then provide detailed answers to Reviewers’ comments.

**Main changes:**

**General:** the English have been revised throughout the whole manuscript. A new figure was added; the figures have been re-numbered and re-uploaded.

**Title:** the title has been modified according to Reviewer #1 in order to better focus on the aim of the study. Consequently, the new title is: “Sensitivity to gait improvement after levodopa intake in Parkinson’s disease: a comparison study among synthetic kinematic indices”.

**Abstract:** we integrated a brief explanation concerning the OFF and ON (medication) state of the individuals affected by Parkinson’s disease.

**Introduction:** according to the Reviewers, missing information have been updated in order to enhance the study background. The aims have been improved removing unnecessary information.

**Methods:** following Reviewers’ observation, methods have been updated to better clarify our study design and intervention. Subject’s data table have been included in the correct section.

**Results:** the table containing subjects’ data has been removed from this section.

**Discussion:** limitations have been added and the conclusions have been moved to a separate section.

**Review Comments to the Author**

**Reviewer #1:** In this study authors aimed at investigating the synthetic indices which are representative of gait stability and harmony in patients with Parkinson’s disease. In particular they focused on their sensitivity in discriminating the OFF and ON conditions. The paper is interesting and the obtained results could be useful to quantify the severity of motor impairment during gait in patients with Parkinson’s disease, but the following minor issues need to be addressed.

**1.Reviewer:** The English in the present manuscript requires an improvement. Please carefully proof-read spell check to eliminate grammatical errors. Some periods need also to be revised, like for example the following ones, in the “Introduction” section:

“Gait analysis (GA) is a widely used methodologies to objectively investigate the gait features in health [5] and both non-motor [6,7] and motor diseases, including PD [3,8–11].”

“However, these studies were carried out regardless of the ON or OFF condition and any the comparison between the two states has been performed.”

**1.Authors:** Thank you for this suggestion. A grammar check has been carried out and errors have been corrected throughout the whole manuscript. The two above-mentioned sentences (lines 50-52 and line 99, respectively) have been modified.

**2.Reviewer:** The title should focus on the indices’ sensitivity in discriminating the ON and OFF condition, since it seems to be the main aim of the authors.

**2.Authors:** According to the reviewer's suggestion, the title has been changed to:

“Sensitivity to gait improvement after levodopa intake in Parkinson’s disease: a comparison study among synthetic kinematic indices.”

**3.Reviewer:** It would be useful integrating a brief explanation of the meaning of the ON and OFF condition in the Abstract and in the Introduction for casual readers.

**3.Authors:** thanks for this recommendation. A brief explanation of the OFF and ON conditions has been included in the abstract (lines 23-24) and in the manuscript (lines 44-46), in order to make the paper clearer for casual readers.

**4.Reviewer:** In the Introduction (Lines 48-49), authors state that through the optoelectronic systems it is possible to acquire spatio-temporal and kinematic parameters with high precision and reliability. Please add some references supporting this statement.

**4.Authors:** As requested, we added some references to support our statement (line 56).

New References:

Ceseracciu E, Sawacha Z, Cobelli C. Comparison of markerless and marker-based motion capture technologies through simultaneous data collection during gait: proof of concept. PLoS One. 2014;9(3):e87640.

Fusca M, Negrini F, Perego P, Magoni L, Molteni F, Andreoni G. Validation of a wearable IMU system for gait analysis: Protocol and application to a new system. Appl Sci. 2018;8(7):1167.

Anwary AR, Yu H, Callaway A, Vassallo M. Validity and consistency of concurrent extraction of gait features using inertial measurement units and motion capture system. IEEE Sens J. 2020;21(2):1625–34.

**5.Reviewer:** Line 49: the sentence “However, more recently, to obtain an overall assessment of gait, many studies have turned to analyse more synthetic…” should be moved in a new paragraph since is not directly linked with the previous topic. Which are the systems commonly used to measure the synthetic indices? Please integrate a description in the Introduction.

**5.Authors:** The sentence has been moved as suggested (line 57). In addition, systems that are usually employed to calculate synthetic measures have been included as well (lines 59-60).

**6.Reviewer:** Table I and patients’ characteristics should be moved in the Subjects section.

**6.Authors:** As suggested by the reviewer, the Table I has been moved under the Subject session (line 124)

**7.Reviewer:** Intervention section: Why did authors acquire 4 gait cycles? Could they add some reference or explanation?

**7.Authors:** We thank the Reviewer for the opportunity to better explain the intervention section. Patients were recorded while walking forth and back at self-selected speed, along a 10-meter walkway. In accordance with the literature (references added at line 148), for each subject and in each condition (OFF and ON), we collected the four best trials and for each of these two gait cycles were selected. The two gait cycles were chosen in the central part of recordings where the markers are highly visible, and the gait was not affected by the possible confounding factors related to the trajectory’s changes. Subsequently the average of eight gait cycles data (two gait cycles for four trials) was used to stabilise the outcome and obtain a more reliable result. Intervention section (line 136) has been updated.

New References:

Nelson AJ, Zwick D, Brody S, Doran C, Pulver L, Rooz G, et al. The validity of the GaitRite and the Functional Ambulation Performance scoring system in the analysis of Parkinson gait. NeuroRehabilitation. 2002;17(3):255–62.

Agosti V, Vitale C, Avella D, Rucco R, Santangelo G, Sorrentino P, et al. Effects of Global Postural Reeducation on gait kinematics in parkinsonian patients: a pilot randomized three-dimensional motion analysis study. Neurol Sci. 2016;37(4):515–22.

Galafate D, Pournajaf S, Condoluci C, Goffredo M, Di Girolamo G, Manzia CM, et al. Bilateral foot orthoses elicit changes in gait kinematics of adolescents with down syndrome with flatfoot. Int J Environ Res Public Health. 2020;17(14):4994.

**8.Reviewer:** Some pictures of the setup and acquisition should be integrated within the manuscript

**8.Authors:** Thank you for this suggestion. Figure 1 has been added (line 152) representing the setup, the camera details and the acquisition representation.

**9.Reviewer:** The quality and resolution of the Figures need to be improved.

**9.Authors:** The figures have been re-uploaded with higher quality and a 600dpi resolution (please download it for full quality; preview is low quality only).

**10.Reviewer:** The limitations and conclusions of the study should be integrated in the paper.

**10.Authors:** Thank you for your suggestion. The limitations of the study have been included in the final part of the discussion (lines 372-376), as well as the conclusions paragraph (line 376).

New References:

Cuesta-Vargas AI, Galán-Mercant A, Williams JM. The use of inertial sensors system for human motion analysis. Phys Ther Rev. 2010;15(6):462–73.

D’Antonio E, Taborri J, Mileti I, Rossi S, Patané F. Validation of a 3D Markerless System for Gait Analysis based on OpenPose and Two RGB Webcams. IEEE Sens J. 2021;

**Reviewer #2**: This observational study points out the sensitivity of different synthetic indices with regard to clinical motor changes of people with PD. More specifically, the authors have assessed objectively the gait performance of 21 subjects with PD in both OFF and ON phases considering synthetic indices including the Trunk displacement Index (TDI) which is a parameter implemented by the authors in order to assess the relationship between trunk and Center of Mass oscillations. Moreover, the authors investigated whether there is a relationship between these indices and the level of motor impairment assessed by UPDRS - part III under both conditions. The findings showed the sensitivity of TDI as well as HR (Harmony Ratio) only in AP (antero-posterior) axis and GR (Golden ratio) in all three dimensions to ON and OFF conditions in people with PD, while only the TDI was directly correlated with the clinical status of PD subjects. The workflow is of good quality and the English is well written with some small grammatical and punctuation errors. However, some minor concerns should be considered as detailed below.

Main comments

**1.Reviewer:** The study design is unclear. Was it a retrospective or a prospective study? The period of patients’ recruitment and the date of study approval by the Ethics Committee are not mentioned in the manuscript. Please clarify these points in order to make your study replicable.

**1.Authors:** Thank you for this comment. This is a mainly observational study, because we performed the gait analysis just before and 40 minutes after levodopa medication; this was clarified in the manuscript within the intervention section (line 136). Moreover, we included the date of study approval by the Ethics Committee (line 122) and specified the period of patients’ recruitment (lines 112-113).

Minor comments

**2.Reviewer:** Abstract **-** Line 29. Your conclusion relates to a study completed . The past should be used.

**2.Authors:** Thank you. We changed it, as suggested (line 33).

**3.Reviewer:** Line 42. Please replace “patients’ problems” with other proper terms (i.e: clinical conditions, status, disorders…).

**3.Authors:** Corrected as suggested (line 48).

**4.Reviewer:** Line 44… GA is a…. methodology…

**4.Authors:** Corrected (line 50).

**5.Reviewer:** Line 49. The right term commonly used in GA is: “spatio-temporal” and kinematic parameters.

**5.Authors:** Modified (line 56).

**6.Reviewer:** Line 51. Correct the punctuation error.

**6.Authors:** We checked and corrected the error (line 59).

**7.Reviewer:** Line 52. …individual”s” affected by motor “pathologies”. Please replace with a proper term.

**7.Authors:** Replaced with “movement disorders” (line 61).

**8.Reviewer:** Line 73. Correct the punctuation error.

**8.Authors:** Corrected (line 82).

**9.Reviewer:** Line 86. PD Individual”s” or people with PD (the plural should be used).

**9.Authors:** Corrected (line 97).

**10.Reviewer:** Line 88. When describing the TDI, information about the score interpretation and its clinical importance lacks. A higher value stands for what?

**10.Authors:** We apologise for this missing information. The interpretation has been added (higher TDI values = worse stability) at lines 91-92.

**11.Reviewer:** Line 90. Grammar should be revised.

**11.Authors:** We rephrased the sentence in a more correct and clear way (lines 100-101).

**12.Reviewer:** Line 92. I suggest to rewrite the aim of the study excluding the parts which can be explained in details in the methods (e.g. the number of recruited subjects should be cited in method and results and not among the aim of the study).

**12.Authors:** Thank you for the suggestion. We removed unnecessary information from this section and (hopefully) improved the readability(lines 101-105).

**13.Reviewer:** Line 99. Was it a retrospective or a prospective study? Please, mention the period of patients’ recruitment.

**13.Authors:** Our study is mainly observational. The patients’ recruitment period has been added as well (lines 112-113).

**14.Reviewer:** Line 108. vi) any other …

**14.Authors:** Corrected (lines 118-119).

**15.Reviewer:** Line 110. Please report also the date of study approval by the Ethics Committee.

**15.Authors:** We added the date at line 122.

**16.Reviewer:** Line 114. I suppose you mean straight “path”.

**16.Authors:** Exactly, thank you for noticing it (line 137).

**17.Reviewer:** Intervention - I am wondering how a person with PD can walk 10 meters by 4 gait cycles. This part needs to be clarified. Moreover, can the authors explain if only one trial consisting of four gait cycles were taken into account per condition or a mean of more trial repetitions was considered for the data analysis?

**17.Authors:** We apologise for being unclear. Individuals (PD patients at very early stage of the disease) walked freely back and forth along a 10-metre long straight walkway. They walked at their own speed and preferred cadence, and we recorded the gait cycles along the entire walkway. After finishing the gait recording, we considered the best four trials and selected two gait cycles (for each trial) in the central area of the walkway, where all markers were visible to the cameras and where the gait was not influenced by turns and changes of direction. The results of the 8 gait cycles (2 cycles for 4 trials) were averaged to obtain more reliable data. Then the patient took his medication and we waited for it to make effect in order to repeat the same acquisition while in ON condition.

The explanation has been hopefully improved at lines 145-149 and references have been added.

New References:

Nelson AJ, Zwick D, Brody S, Doran C, Pulver L, Rooz G, et al. The validity of the GaitRite and the Functional Ambulation Performance scoring system in the analysis of Parkinson gait. NeuroRehabilitation. 2002;17(3):255–62.

Agosti V, Vitale C, Avella D, Rucco R, Santangelo G, Sorrentino P, et al. Effects of Global Postural Reeducation on gait kinematics in parkinsonian patients: a pilot randomized three-dimensional motion analysis study. Neurol Sci. 2016;37(4):515–22.

Galafate D, Pournajaf S, Condoluci C, Goffredo M, Di Girolamo G, Manzia CM, et al. Bilateral foot orthoses elicit changes in gait kinematics of adolescents with down syndrome with flatfoot. Int J Environ Res Public Health. 2020;17(14):4994.

**18.Reviewer:** Line 223. Please add that this result is significant. The statistical differences can be also non-significant.

**18.Authors:** Thank you for this comment. We modified it as suggested (lines 255 and 259).

**19.Reviewer:** Line 323. “Summerising” is not in line with the English style of the whole text.

**19.Authors:** We modified it with “In summary” (line 360).
