# Supplementary material for: Sensitivity to gait improvement after levodopa intake in Parkinson’s disease: A comparison study among synthetic kinematic indices
Source: PLoS One. 2022 May 12;17(5):e0268392. doi: 10.1371/journal.pone.0268392 (PMC9098031; doi:10.1371/journal.pone.0268392)
Supplement: Attachment — Submitted filename: Response_to_Reviewers_V2.DOCX [file pone.0268392.s003.DOCX]

Dear Manabu Sakakibara,

please find enclosed a revision of our paper entitled “Sensitivity to gait improvement after levodopa intake in Parkinson’s disease: a comparison study among synthetic kinematic indices” to be considered for publication in Plos One.

We thank the editor and the reviewers for their efforts in evaluating our manuscript. We did our best to follow the received comments, and hope that this revised submission will be adequate for publication.

In what follows, we firstly summarize the main changes, and then provide detailed answers to Reviewers’ comments.

**Main changes:**

**Abstract:** after reviewing the manuscript, the abstract has been updated accordingly.

**Introduction:** according to the Reviewers, the literature has been updated in order to include recent information on the use of synthetic kinematic indices in Parkinson’s disease.

**Methods:** methods have been updated to include a clearer explanation of the recording method, and the calculated parameters.

**Results:** results and table concerning the spatiotemporal parameters have been added. The section on the correlation analysis has been totally rewritten in accordance with the reviewers’ suggestion (Fig 6 and caption changed accordingly).

**Discussion:** comparison with the existing literature has been improved to include more recent literature. New discussion on the spatiotemporal parameters has been included. The discussion on the correlation analysis has been rewritten. Limitations have been enriched in accordance with the concerning expressed by the reviewers.

In addition, we sent an excel file containing main data supporting the results of this study.

**Reviewers’ comments**

**Reviewer #3:**

**INTRODUCTION**

**1.Reviewer**: The introduction is not updated according to the recent scientific literature on the same topic.

**1.Authors:** We are sorry for this missing. An update of the literature has been integrated into the introduction of the manuscript.

**METHODS**

**2a.Reviewer:** Line 145-150. Many acceleration-derived gait indices require a number of strides > 10 to reach good reliability in their calculations (Riva et al., 2014; Kroneberg et al., 2019). In particular, to calculate the harmonic ratios, 20 harmonics and 20 consecutive gait strides at least are recommended (Pasciuto et al., 2017). This is clearly not possible in a 10-meters pathway. How did you manage this issue?

**2a.Authors:** We apologize for being unclear and thank the reviewers for the opportunity to better explain the methods. As specified in the manuscript (lines 160-170), patients were asked to walk back and forth continuously along a 10-meter walkway. This allowed us to make at least six gait recordings, from which it was possible to extrapolate four good trials, each of which contained two gait cycles (REF: Nelson AJ et al. The validity of the GaitRite and the Functional Ambulation Performance scoring system in the analysis of Parkinson gait; Agosti V et al. Effects of Global Postural Reeducation on gait kinematics in parkinsonian patients: a pilot randomized three-dimensional motion analysis study). In total eight gait cycles for subjects and conditions were used for the statistical analysis. We chose to record the walk only when patients walked in the centre of the walkway, avoiding the recording of direction changes, which represent one of the main motor impediments of patients with Parkinson's disease and which could compromise dynamic stability (REF: Walking Along Curved Trajectories. Changes With Age and Parkinson's Disease. Hints to Rehabilitation). We understand the Reviewers' objection regarding the reduced number of gait cycles used for synthetic index analysis, however it must be considered that patients performed the task twice in the same day in two different conditions, before and 40 minutes after taking L-DOPA. The number of records used for our analysis of the synthetic indices represents the only possible trade off to avoid participants fatigue that inevitably would affect the gait kinematics (REF: Fatigue in Parkinson's disease: A systematic review and meta-analysis). However, in accordance with the reviewers' suggestion we wrote that one of the limitations of our study is the number of gait cycles used for the analysis of the synthetic indices.

**2b.Reviewer:** Moreover, in a recent study (Castiglia et al., 2021), HR values in the antero-posterior direction < 1.50 showed to characterize the gait alteration of subjects with PD in ON condition, as respect to healthy subjects. In figure 2, HR values > 4 are reported for subjects with PD. This is probably due to the low number of consecutive recorded strides.

**2b.Authors:** We want to highlight that HR values are actually calculated at the level of the centre of mass (COM). Most of the studies which use the HR parameters, use data collected by accelerometer positioned at the level of the lower trunk (including Castiglia et al., 2021), as it is the closest external anatomical point with respect to the COM (Iosa et al., 2016, Simoni et al., 2021, Iosa et al., 2013, Fusca et al., 2018, Siragy and Nantel, 2018). Through a stereophotogrammetric system it is possible to retrieve information on the position of the COM itself. It is true that when a subject is at rest in a standing posture, the L3-L5 vertebrae position is the closest point with respect to the COM, but during movement, COM position can change this distance, since its position depends on the position of each body element. Hence, we expect that parameters calculated at L3-L5 level would not be of the same size as the ones calculated at COM level.

However, we calculated the antero-posterior HR values at pelvis level to verify the agreement with the results reported in Castiglia et al., 2021. The authors reported antero-posterior HR values equal to 2.00 (+/-0.55) for Hoehn and Yahr (HY) staging = 1; HR AP 1.94 (+/-0.51) for HY staging = 2; HR AP 1.65 (+/-0.34) for HY staging = 3. Our sample included patients with HY staging ≤ 3 and displayed a value of antero-posterior HR equal to 2.02 (+/-0.41). Hence, pelvis level, our results are similar to the ones reported by Castiglia et al., therefore, the difference highlighted by the reviewer may be driven by the different anatomical position took into account.

**3.Reviewer:** Line 226-227. Did you perform the correlation analysis between the punctual values before and after the levodopa intake, or did you perform it between the improvements (the differences between the ON phase and the OFF phase)?

**3.Authors**: This is an excellent observation. We performed the correlation using the punctual values corresponding to the OFF and the ON conditions. In the new revised manuscript, in accordance with the expressed concerns and suggestions, we performed the correlation between the improvements (i.e., the differences between the ON phase and the OFF phase). The previous correlation analysis has been removed from the manuscript. Fig 6 and caption have been updated accordingly. Methods updated at L260-261.

**RESULTS**

**3.Reviewer:** In the results section, no information on the spatio-temporal parameters have been provided. Since spatio-temporal gait parameters, such as gait speed and step length, are known to improve after levodopa intake (Bryant et al., 2011; Smulders et al., 2016; Baudentistel et al., 2021), spatio temporal parameters should be reported to improve the interpretability of the results.

**3.Authors:** Thanks for this recommendation. In order to facilitate the readability and the interpretability of the manuscript, we reported the information concerning spatiotemporal parameters. Mean, standard deviation, and statistical comparison results (p-value, test statistic and effect size) were reported for: speed, step length, stance time, swing time, cycle time, double support time. Methods updated at L191-195. Results updated at L270-282. Discussion updated L355-359.

**4.Reviewer:** As regard the correlation analysis, if the aim of the study was to observe the sensitivity-to-change of the indices after the levodopa intake, a correlation analysis between the improvements in the kinematic and the clinical variables should be reported.

**4.Authors:** As aforementioned, following the suggestions, we performed a correlation analysis between the improvements of the kinematic indices and the clinical variable (UPDRS). Results reported at L323-346.

**5.Reviewer:** Moreover, since UPDRS improvements are correlated with the improvements in gait speed after levodopa intake, it would be relevant to exclude the effects of the gait speed improvements between in the correlation analysis.

**5.Authors:** We improved our analysis by repeating the correlation test between synthetic indices improvement and clinical improvement, controlling for the effect of the gait speed. Even in this case, the correlation between the TDI improvement and the UPDRS improvement resulted to be statistically significant. Methods updated at L262-263. Results updated at L323-346.

**DISCUSSION**

**6.Reviewer:** L 304-306. I believe that this paragraph needs to be improved. I don’t really believe that the authors assessed the sensitivity or the ability to discriminate between on and off conditions. To do that, the authors should have performed other statistical analysis, such as Mann-Whitney test using the medication condition as between factor, AUC calculations, sensitivity and specificity calculations at the optimal cutoff points the discriminate between the two phases. I believe that, in this study, the Authors have rather observed the responsiveness to levodopa intake of the synthetic kinematic indices, in terms of sensitivity-to-change. For this purpose, the authors should focus on the effect size values, to describe the magnitude of the modifications after the levodopa intake.

**6.Authors:** Thanks for this highly pertinent observation. We have used the term “sensitivity” absolutely incorrectly. As the reviewer rightly states, in our work we evaluate the responsiveness to levodopa of the kinematic synthetic indices under consideration. Accordingly, we revised the discussion section.

**7.Reviewer:** L352-354. The authors should explain the reason for using another method than the one used by Iosa et al.

**7.Authors:** We reported the explanation at lines 417-423. We wanted to observe how much each individual golden ratio (GR) was far from phi. Using the Iosa et al. method, a group of subjects could present a mean GR extremely close to phi, but the specific values of GR of each individual are very far from phi. Conversely, another group of people may have a mean GR less close to phi compared to the previous hypothetical group, but in this case each subject presents an individual GR close to phi. Paradoxically, the first group would result to have the best GR value (with higher standard deviation of course), while the truth is that the second group presents values closer to phi. However, the main issue occurs when performing correlation tests. Performing a linear correlation would not be correct in this case because the individuals are logically expected to show worse condition (high UPDRS) when GR is very low compared to phi, better condition (low UPDRS) when GR is close to phi, and again worse condition (high UPDRS) when GR is very high compared to phi. In this case the relationship between variables should be quadratic and cannot be represented by a linear correlation. The way we calculated the GR values, as showed in the method section allows to use linear correlation tests.

**8.Reviewer:** L 354-356. This information is not reported in the results section nor in the methods section. Consider adding it in the previous sections or removing the sentence from this section.

**8.Authors:** We updated the methods (L243-244) and the results (L333-335) section in order to report the described result.

**9.Reviewer:** L357-358. Please, explain which differences in the subjects’ characteristics could have led to the differences between your study and the one from Iosa et al.

**9.Authors:** We reported the information as requested in L426-428.

**10.Reviewer:** Again, the discussion is not based on the previous literature on the same topic (indexes of gait stability in Parkinson disease).

**10.Authors:** We updated the discussion with more recent literature concerning the use of synthetic gait indices in Parkinson’s disease.

**Reviewer #4:**

**Reviewer:** I enjoyed reading this revised version of the manuscript. I think the authors did a good job addressing the useful reviewers' comment.

**Authors:** We are glad the reviewer enjoyed the reading of the manuscript. We thank the reviewer for his effort.
